# Physician Rating Websites: What Aspects Are Important to Identify a Good Doctor, and Are Patients Capable of Assessing Them? A Mixed-Methods Approach Including Physicians’ and Health Care Consumers’ Perspectives

**DOI:** 10.2196/jmir.6875

**Published:** 2017-05-01

**Authors:** Fabia Rothenfluh, Peter J Schulz

**Affiliations:** ^1^ Institute of Communication and Health Department of Communication Sciences Università della Svizzera italiana Lugano Switzerland

**Keywords:** physician rating websites, quality of care, health care consumers, judgment, physicians, doctors, Delphi technique, cross-sectional study, ethics, assessment, electronic word of mouth

## Abstract

**Background:**

Physician rating websites (PRWs) offer health care consumers the opportunity to evaluate their doctor anonymously. However, physicians’ professional training and experience create a vast knowledge gap in medical matters between physicians and patients. This raises ethical concerns about the relevance and significance of health care consumers’ evaluation of physicians’ performance.

**Objective:**

To identify the aspects physician rating websites should offer for evaluation, this study investigated the aspects of physicians and their practice relevant for identifying a good doctor, and whether health care consumers are capable of evaluating these aspects.

**Methods:**

In a first step, a Delphi study with physicians from 4 specializations was conducted, testing various indicators to identify a good physician. These indicators were theoretically derived from Donabedian, who classifies quality in health care into pillars of structure, process, and outcome. In a second step, a cross-sectional survey with health care consumers in Switzerland (N=211) was launched based on the indicators developed in the Delphi study. Participants were asked to rate the importance of these indicators to identify a good physician and whether they would feel capable to evaluate those aspects after the first visit to a physician. All indicators were ordered into a 4×4 grid based on evaluation and importance, as judged by the physicians and health care consumers. Agreement between the physicians and health care consumers was calculated applying Holsti’s method.

**Results:**

In the majority of aspects, physicians and health care consumers agreed on what facets of care were important and not important to identify a good physician and whether patients were able to evaluate them, yielding a level of agreement of 74.3%. The two parties agreed that the infrastructure, staff, organization, and interpersonal skills are both important for a good physician and can be evaluated by health care consumers. Technical skills of a doctor and outcomes of care were also judged to be very important, but both parties agreed that they would not be evaluable by health care consumers.

**Conclusions:**

Health care consumers in Switzerland show a high appraisal of the importance of physician-approved criteria for assessing health care performance and a moderate self-perception of how capable they are of assessing the quality and performance of a physician. This study supports that health care consumers are differentiating between aspects they perceive they would be able to evaluate after a visit to a physician (such as attributes of structure and the interpersonal skills of a doctor), and others that lay beyond their ability to make an accurate judgment about (such as technical skills of a physician and outcome of care).

## Introduction

### Electronic Word of Mouth in Health Care

The Internet has created a space for interaction where anyone with a Web connection can freely voice his or her opinion on any conceivable subject. One special feature of this public sphere is rating platforms that allow consumers to review goods and services purchased on and offline [1]. This has put power into consumers’ hands who can instantly issue their commendation or crushing verdict to a mass audience. Using electronic word of mouth (eWOM) when purchasing a new good or service, comparing features, prices, and reviews has become commonplace for many consumers [2]. They especially appreciate recommendations from former clients to inform their choice of the so-called experience or credence goods and services [3,4], where consumers lack expertise or experience [5]. For certain industries such as tourism or gastronomy, where this development appears to be largely accepted, receiving positive Web-based recommendations through eWOM has become crucial to stay in business (see [6]).

This development has also found its way into health care. The so-called physician rating websites (PRWs) are numerously available either as separate rating websites exclusively for physicians, as part of a larger review platform where also other goods and services can be appraised, or even as part of a hospital’s staff directory website. This culture of reviewing in health care developed in parallel to a change in the relationship between patients and physicians. The traditionally paternalistic rapport between the doctor and patient has transformed into a more patient- or client-centered approach, whereby passive patients have become active health care consumers [7,8].

This change in the doctor-patient–relationship was also backed by initiatives and corresponding research efforts. Patient empowerment [9] has become a globally fostered goal through charters founded by patient organizations [10] or conferences held by the WHO [11]. Simultaneously, concepts to foster patients’ involvement in the health care process, such as shared decision-making with the physician [12,13], patient self-efficacy [14], and autonomy [15] have been promoted in research and practice to demolish the traditionally hierarchical relationship between a physician and a patient.

The movement away from a paternalistic model to a more egalitarian collaborative encounter has changed today’s relationship between a physician and a patient [16]. PRWs take this movement to the next level: no longer are physicians and patients collaborative entities, but the latter is becoming a consumer of the former’s service with the power to publicly review and assess the health care service received. The traditional paternalistic relationship between the provider and the consumer [8,12,17] and a vast knowledge gap between the two parties created by professional training and experience [18] led health care providers to voice skepticism toward ratings of hospitals and physicians [19].

Health care providers worry about unjustified negative reviews [20-22] because physicians’ efforts to get negative reviews on PRWs deleted are rarely successful. Suing review platforms may even gain more public attention than keeping a negative review on one’s profile [23]. Hence, some doctors who were reviewed negatively even breached their oath to treat patient information with confidentiality by leaking private details about the patients’ care when replying to comments on PRWs [24,25].

Despite the debatable nature of physician rating websites due to the anonymity of the posted reviews, the low number of reviews per physician, and the lacking legal regulations [20,23,26], patients keep reviewing their doctors. Simultaneously, PRW users continue to read reviews and consider them in their choice of a health care provider [27,28]. The access to PRW reviews is a two-edged sword; it may reveal transgressions and ineptitude of health care providers that may have stayed in the dark otherwise, but could also make unjustified malpractice allegations to providers just because the consumer lacks knowledge to evaluate the health care encounter accurately.

Experts and researchers pose a different perspective, arguing that PRWs could aid the creation of more transparency and surveillance of the health care system [29,30]. PRWs could, for example, provide instant feedback if quality of care is alarmingly deteriorating in a certain care facility [31]. A study with German physicians on their use of PRWs showed that this may bear some fruit; more than half of the participating doctors used PRWs for the improvement of their quality of care, particularly to ameliorate their communication with the patient and the scheduling of appointments [32]. Given the tension between advocates and opponents of PRWs, research is needed to address from both physicians’ and health care consumers’ perspectives how the quality of a physician and the treatment he or she provides could be operationalized into sensible indicators for physician rating websites.

### Theoretical Background

Defining and measuring quality of care has a long history due to its complexity. The World Health Organization describes quality in health care as effective, efficient, accessible, acceptable, patient-centered, equitable, and safe [33]. Due to its latent qualities, scholars have developed various models to operationalize the concept of quality care. Donabedian, a prominent scholar in the field, divides quality of care into pillars of structure, process, and outcome [34-36]. According to Donabedian, “structure denotes the attributes of the setting in which care occurs” (p. 1745), such as material and human resources and the organizational structure [35]. The process of care is concerned with the way care is provided, such as how a diagnosis is made or a treatment is executed by the health care provider [35]. The third pillar, outcome of treatment or care encounter, denotes the effects that the treatment has had on the patient such as the improvement in health status or a change in patient’s behavior toward a cure-inducing lifestyle [35].

The Donabedian model is analogous to the division Maribito suggested in his categorization of health care into aspects of search, experience, and credence properties [37]. In health care, search aspects could be translated into features of infrastructure of the practice facilities as they are transparently comparable even before a physician visit. The treatment process and outcomes of the received care could either be categorized as experience or credence aspects; the experience of a treatment encounter may allow health care consumers to assess some aspects such as the interpersonal demeanor of a physician. Other aspects of more technical nature may not even be assessable after a health care consumer experienced them, making them credence traits. The application of a search, experience, and credence model to health care is not novel [37]. However, how the model on search, experience, and credence could concretely be translated into the assessment of physicians has not yet been answered. Hence, the question emerges which aspects of care can be evaluated and whether physicians and health care consumers agree on that. In order to answer these questions, samples of experts (physicians) and health care consumers were studied. This study’s objectives are summarized in the following research questions: What aspects do physicians consider to be important to identify a good physician? To what degree do health care consumers agree with the indicators suggested by physicians to identify a good physician? and In which aspects or dimensions and to what extent do physicians and health care consumers agree that these aspects can be evaluated by patients after a physician visit?

## Methods

### Step 1: Delphi Study With Physicians

This study was divided into two parts: a Delphi study with physicians and an electronic questionnaire with adult health care consumers (see Multimedia Appendices 1 and 2). Before the study launch, ethical approval was obtained by the ethics committee of the Università della Svizzera italiana (CE 2015-8).

The Delphi study was conducted over two rounds consulting guidelines published by von der Gracht [38]. The sample comprised Swiss physicians from 4 different specializations (general physicians, pediatricians, orthopedic surgeons, and dentists). These specializations were chosen by the research team due to the diversity of services and treatments they provide, the distinct skills needed in these specializations, the different audiences served, and because some of the treatments these doctors offered are not covered by basic health insurance in Switzerland (dentist visits are not covered). First, indicators of quality of care were theoretically derived from the Donabedian quality of care model and work that extended his classification [35,39], pretested with 6 physicians and adapted where necessary. This yielded 43 indicators on the basis of the quality of care dimensions (1) structure (infrastructure and staff at the hospital or practice), (2) process (technical and interpersonal skills of the physician), and (3) outcome of care.

In the first round of the Delphi study, doctors were asked to rate each of these indicators twice: (1) how important that indicator was to identify a good physician of his or her specialization (1=not at all important; 5=very important), and (2) how well a patient could evaluate this quality of care indicator after a first physician visit (1=not at all; 5=very well). After each section of the survey, doctors were provided with a blank space to comment on the questioned indicators and to add new indicators that were not included in the questionnaire yet. Data for the first round was collected from October to December 2015.

A total of 120 physicians were invited via email (with information about the study and a link to the survey) or through a collaboration partner at the Central Switzerland Physician Association to participate in the Delphi study. Of the total, 29 physicians consented to participate and all joined in the first Delphi round. Nineteen physicians completed the second round as well. The sample in the first round consisted of 9 general practitioners (33.3%), 5 pediatricians (17.9%), 6 orthopedic surgeons (21.4%), 7 dentists (25.0%), and 1 internist (3.6%). Participants were predominantly male (n=19, 66.6%), aged between 29 and 63 years (mean 47.6 years, SD 9.6 years), and had on average 19.6 years of work experience (range 3-37 years, SD 10.1 years).

The results from the first round of the Delphi questionnaire were analyzed using SPSS statistical software 23.0 (IBM Corp). We consulted guidelines published by von der Gracht [38] to define decision criteria to establish whether participants reached agreement. The decision criteria yielded the following categorization: (1) indicators to which a two-third majority (66.66%) or more of physicians agreed that they were important (scored 4 or 5 on the 5-point scale); (2) indicators, which at least two-thirds (66.66%) scored as unimportant (ie, scores of 1 or 2); and (3) remaining indicators that were either scored mainly on a middle value (ie, 3) or highly scattered. Indicators categorized in the second category were excluded from the second Delphi round (n=5), whereas the ones categorized into groups 1 and 3 were adjusted or rephrased where necessary and presented again (category 1 for confirmation and stability and category 3 for clarification). For each of the debated indicators, the modes and corresponding percentages of votes of the first round’s results were presented to the participants. Physicians were asked to choose the one value among the results from round 1 that they most agreed with. They were given free text space to explain their responses. The same logic and classification criteria were applied to the answers about whether these indicators can be evaluated by patients after a first visit. Data for the second round of the Delphi study was collected between March and April 2016. The results of the second Delphi round were analyzed using the same criteria and classification listed above (see Multimedia Appendices 1 and 2). Physicians reached agreement on 35 indicators applying the same criteria as after round 1. Stability after two rounds was reached for 32 indicators in terms of importance, and for 27 indicators in terms of evaluation by health care consumers.

### Step 2: Cross-Sectional Survey With Health Care Consumers

Subsequently, a cross-sectional e-questionnaire with health care consumers was launched surveying the same indicators that were retained for the second round Delphi questionnaire (see Multimedia Appendices 1 and 2). Participants were asked to rate on 5-point scales (1) how important the listed indicators were for them to identify a good physician and (2) to what extent they would feel capable of evaluating an indicator of care after the first visit with a physician. The questionnaire was pretested over 3 rounds with 5-10 participants each. Survey design and layout features were adjusted where necessary.

The survey was launched via Qualtrics between April and June 2016 via snowball sampling through email and social media. Eligible for participation were individuals who were (1) 18+ years old, (2) residing in Switzerland, (3) and had Internet access. A total of 211 participants completed the survey. Participants were predominantly female (63%), on average 38.24 years old (range 19-74 years, SD 15.51), and the vast majority was of Swiss nationality (188/211, 89.1%). The sample was well-educated including 53.9% respondents with some form of tertiary education (PhD: 12/211, 5.7%; university degree: 74/211, 35.1%; applied science university degree: 29/211, 13.7%; high school degree: 33/211, 15.6%; apprenticeship: 55/211, 26%; secondary school: 3/211, 1.4%; and other: 5/211, 2.4%).

The vast majority of participants had already used review websites (82.9%), with Tripadvisor being the best known and the most used (66.4%), followed by Booking (58.8%), Amazon (49.8%), and Ricardo (48.8%). The majority of health care consumers had a neutral opinion toward physician rating websites (48.8%), whereas 36.0% were in favor of being able to write and access physician ratings online and 15.2% were opposed. Only about a tenth of participants worked in a medical profession (19/211, 9.0%).

### Step 3: Comparison Between Physicians’ and Health Care Consumers’ Perceptions

In a third step, agreement between physicians and health care consumers was calculated. The results of the second round of the Delphi study and the electronic health care consumer survey were ordered and categorized for (1) importance and (2) health care consumers’ evaluation capability. First, the health care consumer data were cleaned. Means, medians, and standard deviations were computed. Then, for the purpose of categorization, all indicators were recoded from 5- to 3-point scales (1 and 2=not important or not evaluable; 3=unsure or debatable; 4 and 5=important or evaluable). If the following decision criteria were met, the indicator was categorized to be important: (1) if at least two-thirds (66.66%) of the sample voted for an indicator to be important or very important, (2) if criterion 1 was not met, the mean of the indicator on the 5-point scale had to be above 3.5 and the standard deviation less than 1. The same decision rules were applied to assess whether an indicator was evaluable by health care consumers. The first criterion was adapted from the Delphi study, whereas the second criterion was constructed to assert that the opinions did not diverge much.

On the basis of those results, indicators were ordered into 1 of 4 categories for both the physician sample and the health care consumer sample: (1) indicator is important and can be evaluated, (2) indicator is important but cannot be evaluated, (3) indicator is not important but can be evaluated, and (4) indicator is not important and cannot be evaluated by health care consumers. Indicators were ordered into a 4×4 matrix with physicians’ assessment represented on the horizontal and health care consumers’ on the vertical dimension (see Table 1). We then calculated the agreement on importance and evaluation capability of health care consumers based on the physicians’ and the health care consumers’ ratings.

## Results

### Delphi Study and Indicator Development

The Delphi study yielded 35 indicators that fulfilled the consensus criteria derived from the literature (see [38]). The comments from physicians were worked into the analysis and provided additional insight into physicians’ perceptions, some of which are listed later in this section. Physicians unanimously agreed that all indicators of interpersonal competence were very important. The technical aspects of care and the outcome indicators were also rated highly, but the overall agreement was lower.

Infrastructure however was overall assessed to be slightly less important; especially aspects of practice management and competence of the medical assistants were deemed less relevant. The physicians who scored the management and staff less important commented that from their point of view, practice organization and particularly the quality of the staff and medical practice assistants did not provide information about the quality of a physician. They argued that the quality of the physician, the quality of his staff, and the practice management should not be mixed up. From their point of view, the quality of a physician was independent of the other listed aspects. Hence, they scored these indicators to be less relevant than some of their colleagues who perceived components of management, staff, and infrastructure as integral aspects to recognize a good physician.

Four indicators, namely, efficiency, hygiene standards, patient satisfaction, and the presentation of an appropriate number of treatment options were not unanimously voted to be important. Specifically mentioned was that TARMED, the mandatory Swiss cost calculation system, specifies the rate of ambulatory treatments to standardized levels in Switzerland (see [40]) and thus regulates efficiency by law. Also, respecting hygiene standards was not unanimously accepted as a quality standard from doctors’ perspective because hygiene is a prerequisite for any practice in Switzerland as they have to pass quality assessments (such as EQUAM [41] or QBM [42]) to stay open. Also patient satisfaction, an outcome measure, did not achieve undivided agreement among doctors. Physicians voiced concerns that this measure could not necessarily be an end goal, especially if the patient requested a treatment that was not justifiable by best practice guidelines. Hence, they deemed patient satisfaction a double-edged sword: important but not at the cost of a correct treatment. Furthermore, doctors stated that offering a patient an appropriate number of treatment options may overwhelm some health care consumers. If a patient was overstrained with many options, the quality of the decision would be lower.

### Agreement Between Physicians and Health Care Consumers on Importance and Evaluation Capability of Care Aspects

Health care consumers’ answers on which indicators are important to identify a good physician and on whether these are evaluable after the first visit were compared with the answers of the experts (physicians). The results for both dimensions are listed in detail for both health care consumers and physicians: Multimedia Appendix 1 shows how important the indicators were scored by the two samples, and Multimedia Appendix 2 presents the results on health care consumers’ capability to assess those aspects after the first care encounter. Indicators are listed according to the Donabedian model: Structure (1 infrastructure, 2 staff, 3 organization), process (4 technical skill, 5 interpersonal skill), and outcome of care (6 outcome). All indicators were classified into a 4×4 matrix in order to visualize agreement between physicians and health care consumers (see Table 1).

On the basis of our coding, 26 out of 35 indicators were assessed the same way by health care consumers and physicians (Table 1). Agreement between the two parties according to Holsti’s method [43] was calculated at 0.7429 if the assessments of importance and evaluability are both considered together. As this number is larger than 0.70, the agreement between physicians and health care consumers is fairly high.

Looking at the data more closely, the aspects of physician performance on which both doctors and health care consumers agreed that they were both important and evaluable by health care consumers included: infrastructure, organization, and management of the practice, quality, education of, and collaboration among the staff, and interpersonal demeanor of the doctor. Physicians and health care consumers also agreed on 8 aspects that were assessed by both parties as important, but not evaluable by health care consumers after the first visit. Mainly, the technical ability and skills of the physician (such as the way the physician made the diagnosis, whether treatment steps were correctly executed, and whether the doctor follows hygiene guidelines) and outcome measures (for example, whether the treatment was efficient) were assessed important, yet not assessable by health care consumers.

Disagreement between physicians’ and health care consumers’ judgments primarily emerged concerning health care consumers’ capability to assess certain medical components of care. Health care consumers differed from physicians in thinking that they were not able to tell whether they were diagnosed correctly, whether the diagnosis was initiated timely and treatment started swiftly, and whether their concerns were treated confidentially. Physicians however judged health care consumers as incapable of assessing whether the necessary diagnostic instruments were available in the practice and if the doctor already had a lot of work experience.

Investigating the different dimensions of care by calculating the overall means, the results of the health care consumers show that they judged the technical skills of the physician to be the most important (mean 4.70, SD 0.37), followed by the interpersonal skills (mean 4.64, SD 0.38), and outcome of care (mean 4.46, SD 0.48). Infrastructure (mean 4.20, SD 0.56), organization (mean 4.00, SD 0.72), and the quality of the staff (mean 3.89, SD 0.57) were judged to be less important.

In terms of health care consumers’ self-perceived capability to assess a physician, they attributed themselves the highest competence to assess the organization and management of the practice (mean 4.17, SD 0.79), followed by a physician’s interpersonal skills (mean 4.09, SD 0.66), and the infrastructure and accessibility (mean 4.07, SD 0.67). The outcome of care (mean 3.85, SD 0.74), the quality of the staff (mean 3.70, SD 0.63), and technical skills of the doctor (mean 3.46, SD 0.79) were perceived to be more difficult to judge.

**Table 1:** A 4×4 matrix classification of indicators denoting importance to identify quality of health care and health care consumers’ evaluation capability.

Indicators^a^		Physicians			
		Important and evaluable	Important but not evaluable	Not important but evaluable	Not important and not evaluable
Health care consumers	Important and evaluable	1.4, 2.2, 2.5, 3.1, 3.2, 3.3, 3.4, 4.5, 5.1, 5.2, 5.3, 5.4, 5.5, 5.7, 5.8, 6.1, 6.2, 6.3	1.2, 2.4		
	Important but not evaluable	4.1, 4.9, 5.6	2.6, 4.2, 4.3, 4.4, 4.6, 4.7, 4.8, 6.4		
	Not important but evaluable	1.1, 1.3, 2.1			
	Not important and not evaluable		2.3		

^a^The indicators that were categorized into the graph above are numerically listed in Multimedia Appendices 1 and 2.

### Patients’ Ratings of Their Ability to Evaluate Aspects of Health Care by Gender and Educational Level

Independent sample means *t* tests [44] were conducted to check whether there were differences in the health care consumer sample based on the sociodemographic variables. As we conducted multiple tests, we applied the Holm-Bonferroni method and report sequential corrected *P* values (at alpha=.05) to control for type-1 error [45,46]. Only significant results were reported (see Tables 2-5). In terms of gender, women scored 7 aspects of care higher or more important to identify a good physician than men (Table 2). Women scored aspects such as privacy, cleanliness and hygiene, information provision, the presentation of treatment options, and empathy to be more important than men did. Also, in terms of self-perceived capability to assess aspects of care, women scored higher than men in one aspect of care. Namely, women perceived to be better capable of assessing cleanliness and hygiene. Overall, this leads to the conclusion that women generally assign higher scores than men both in terms of importance and tend to have a slightly higher self-perceived capability to assess aspects of care.

**Table 2:** Gender differences in aspects that were deemed important by health care consumers (independent samples *t* tests). Standard deviations appear in parentheses below means.

Indicator		Gender			
		Male	Female	*t*	*df*
1.3	The patient’s privacy is guaranteed	4.24 (0.91)	4.67 (0.68)	−3.569^a^	127.56
1.4	The practice is clean and hygienic	4.58 (0.64)	4.86 (0.39)	−3.630^b^	110.78
4.4	The physician presents an appropriate and complete number of treatment options to the patient	4.31 (0.84)	4.69 (0.54)	−3.616^b^	114.59
4.5	The physician assesses the patient’s handicaps correctly and presents him or her with appropriate information and treatment options	4.35 (0.77)	4.62 (0.60)	−2.740^a^	131.51
4.7	The physician and his team adhere to hygiene guidelines	4.63 (0.65)	4.89 (0.34)	−3.280^a^	102.49
5.3	The physician comprehensibly communicates all important information about the diagnosis and treatment	4.71 (0.51)	4.87 (0.36)	−2.644^a^	126.01
5.7	The physician treats the patient empathically	4.29 (0.80)	4.63 (0.60)	−3.255^a^	128.52

^a^*P*<.05.

^b^*P*<.001.

To assess if individuals with differing educational levels have divergent opinions on what aspects of care are important to identify a good doctor and whether they are assessable by health care consumers, the sample was subdivided into 2 groups. Individuals who completed tertiary education (ie, PhD, university, and applied science university) were denoted as “high education,” whereas individuals without tertiary education (ie, high school, apprenticeship, and secondary school) were classified as “low education.”

**Table 3:** Gender differences in health care consumers’ self-perceived capability to evaluate a doctor (independent samples *t* tests). Standard deviations appear in parentheses below means.

Indicator		Gender			
		Male	Female	*t*	*df*
4.7	The physician and his team adhere to hygiene guidelines.	3.38 (1.13)	3.90 (1.09)	−3.279^a^	209

^a^*P*<.05.

The results of independent samples *t* tests show that individuals with lower education scored 9 items as significantly more important than individuals with higher education (eg, experience and friendliness of staff, correct execution of treatment steps, empathy, and patient involvement in the treatment process). When asked about their self-perceived ability to assess aspects of care after the first visit, individuals with lower education perceived themselves to be significantly better capable of assessing whether a timely diagnosis was made and the treatment swiftly initiated, as well as whether their concerns were treated confidentially. Participants with a higher educational background only scored significantly higher than individuals with low education in terms of capability to assess if decisions about the course of action were made in collaboration between the physician and the patient.

**Table 4:** Educational differences in terms of importance (independent samples *t* tests). Standard deviations appear in parentheses below means.

Indicator		Educational level			
		Low	High	*t*	*df*
2.2	The medical practice assistants are helpful and friendly	4.54 (0.56)	4.23 (0.95)	2.941^a^	190.624
2.3	The medical practice assistants are experienced in their work	3.55 (1.00)	3.22 (1.01)	2.355^a^	204
2.5	The physician collaborates well with his team	4.51 (0.57)	4.18 (0.80)	3.256^a^	204
3.4	The physician is available for phone consultations (before or after the appointment)	4.23 (0.79)	3.86 (0.99)	2.906^a^	204
4.6	The physician and his team execute the treatment steps correctly	4.82 (0.41)	4.11 (0.61)	2.533^a^	199.250
5.5	The physician motivates the patient to actively take part in the treatment process	4.52 (0.64)	4.22 (0.88)	2.831^a^	202.718
5.7	The physician treats the patient empathically	4.66 (0.62)	4.38 (0.73)	2.938^a^	203.110
6.2	The patient is satisfied with the treatment.	4.74 (0.491)	4.49 (0.754)	2.863^a^	197.191
6.3	The patient returns to the same physician for check-ups, etc (patient loyalty).	4.62 (0.491)	4.49 (0.754)	3.266^b^	203.841

^a^*P*<.05.

^b^*P*<.001.

**Table 5:** Educational differences in terms of evaluation capability (independent samples *t* tests). Standard deviations appear in parentheses below means.

Indicator		Educational level			
		Low	High	*t*	*df*
4.9	The physician makes the correct diagnosis timely and initiates the treatment swiftly	3.58 (1.08)	3.22 (1.02)	2.485^a^	204
5.6	The patient’s concerns are treated confidentially	3.22 (1.26)	2.81 (1.40)	2.183^a^	204
5.8	Decisions about the course of action are made together with the patient	4.14 (1.03)	4.43 (0.76)	−2.261^a^	161.254

^a^*P*<.05.

^b^*P*<.001.

**Table 6:** Pearson’s correlation coefficient on importance of indicators to identify a good physician and age (N=211).

Indicator		Age
2.3	The medical practice assistants are experienced in their work	.241^b^
2.4	The physician has a lot of work experience and practices already for a longer time	.214^b^
3.1	The waiting time until the next available appointment is short	.238^b^
3.2	The patients are notified in case of appointment delays or cancellations	.151^a^
3.3	It is easy to schedule an appointment with the physician	.154^a^
5.5	The physician motivates the patient to actively take part in the treatment process	.150^a^
5.8	Decisions about the course of action are made together with the patient.	.148^a^

^a^*P*<.05.

^b^*P*<.001 (2-tailed).

### Health Care Consumers’ Assessment of Importance and Perceived Capability to Evaluate a Physician Based on Age

The health care consumer data were further analyzed to check whether there is a relationship between age and the importance health care consumers’ attribute to certain aspects of care and their self-perceived capability to assess health care providers. The results from the correlation analysis reveal that with older age health care consumers perceive aspects of organization and shared decision making as more important (Table 6). At the same time, the older health care consumers are, the more they perceive aspects of care assessable, particularly the organization of the practice, the physicians’ technical competency (eg, correct diagnosis and treatment execution, timely diagnosis, etc), as well as the efficiency of the treatment (Table 7).

**Table 7:** Pearson’s correlation coefficient on health care consumers’ perceived capability to assess aspects of health care and age (N=211).

Indicator		Age
1.1	The physician’s practice is easily accessible and reachable by public transport and car	−.149^a^
2.3	The medical practice assistants are experienced in their work	.257^b^
2.4	The physician has a lot of work experience and practices already for a longer time	.179^b^
2.5	The physician collaborates well with his team	.223^b^
4.1	The patient is diagnosed correctly	.202^b^
4.2	The physician asks the relevant questions and orders the correct tests to reach the correct diagnosis	.189^b^
4.3	The physician proceeds systematically and competently to reach the correct diagnosis	.168^a^
4.7	The physician and his team execute the treatment steps correctly	.224^b^
4.9	The physician makes the correct diagnosis timely and initiates the treatment swiftly	.173^a^
5.8	Decisions about the course of action are made together with the patient	.171^a^
6.4	The treatment was efficient (ie, cost-benefit ratio was accurate)	.142^a^

^a^*P*<.05.

^b^*P*<.001 (2-tailed).

## Discussion

### Principal Findings

Our results show that a majority of health care consumers and physicians categorized 26 out of 35 indicators similarly in terms of both importance to identify a good doctor and patients’ perceived competence to evaluate them after the first visit. The data show that the majority of indicators were assessed to be both important and able to be evaluated by health care consumers, thereby creating limited variance. This occurred because indicators that physicians agreed were not important in the first round of the Delphi study and were excluded in the second round. Also, these items were not presented to health care consumers, which may explain some of this lacking variance. Nevertheless, the data provide an initial indication that health care consumers have a moderate self-perceived ability to assess the quality and skill of a medical doctor.

Looking more closely into the care aspects that are deemed assessable, we found that health care consumers and physicians judged the formers’ ability to evaluate the infrastructure, organization, and physician’s interpersonal behavior to be high. These aspects of quality of care lend themselves for evaluation by health care consumers because they do not require medical expertise to be assessed. In terms of aspects that cannot be assessed by health care consumers, our results showed that health care consumers and physicians had reservations toward patients’ ability to assess a doctor’s technical skills or the outcome of care. Specifically, the quality of a physician’s education, the process of reaching the correct diagnosis, the execution of the treatment (competence, hygiene, and efficiency), and the presentation of treatment options was mutually accepted to be crucial but not assessable by health care consumers.

These results suggest that health care consumers acknowledge and are aware of the gap in expertise between doctor and patient that arises based on doctors’ medical education [18], even though the numerous ethical concerns previously voiced in literature have suggested otherwise [20,21,47]. However, whether health care consumers would be cautious or even refuse to assess a physician’s competence or technical skill when asked to review a doctor in a real-life remains questionable. An analysis of open-ended textual reviews on a German PRW found that in 63% of the 3000 analyzed cases, PRW users assessed physicians’ competence, a technical aspect of care [48]. Explaining this contradiction, and whether the gap between the intention to review technical aspects of care and the actual reviewing behavior could provide further insights into these incongruent findings, may be the subject of further research.

Physician selection research shows that technical aspects are often identified as the paramount criteria when health care consumers have to select a doctor [49-51]. The results in this study confirmed that health care consumers perceive technical skills of a physician to be the most important to recognize a good physician. Nonetheless, it has been confirmed that most health care consumers are not capable of using and accurately interpreting medical or technical quality of care reports to inform their physician selection [52-54]. Hence, data presentation formats that take into account that health care consumers have difficulty to assess and interpret technical aspects of care, and hence translate quality of care data in an understandable manner, are needed [52,53]. This study suggests that health care consumers are not a good source to provide this kind of information.

The results further show that for 6 of these 26 indicators, which mainly concern the physicians’ competence to reach and execute a diagnosis, should not be reviewed by health care consumers because they lack competence to do so. Mixed information sources to report different aspects of care quality, combining patient reviews as a complement to customary quality reports have already been suggested by Verhoef and colleagues [26]. A large-scale experiment by Schlesinger and colleagues attempted to do that. They presented PRWs featuring quality of care data in combination with written reviews. However, the combined format did not yield better physician selection results, especially if choices grew more complex with larger choice sets and more indicators and information present [55]. Hence, finding PRW formats in which health care consumers can voice their opinion on aspects that are deemed assessable, while condensing and summarizing technical quality of care information in a format that is understandable by health care consumers (as suggested by Hibbard et al [54]) should be the subject for further research.

In the analysis on differences on the perceived importance and evaluation capability based on gender and educational level, two patterns emerged: women and lower educated individuals rate indicators higher or more important and perceive aspects of care as better evaluable than men and individuals with higher educational background. Given that women are more affine toward health issues and search for health information more eagerly [56] and are more aware and likely to use PRWs than males [28], this experience with health information on the web may lead to a perception of expertise. This hypothesis should be tested in future research.

Furthermore, our results suggest that individuals without tertiary education attribute themselves a higher capability to evaluate aspects of health care than individuals with some university degree. This finding is alarming as lower educational levels have been associated with low health literacy [57], more difficulty in processing quality of care information, and less optimal health care choices [55,58,59]. These findings suggest that individuals who are most in need for tools that guide them toward a better search and assessment of Web-based information [60], attribute themselves higher expertise than they probably have from an objective point of view. As the Internet is a resource that may lead to or even encourage dangerous outcomes if guidance is lacking [60], more effort should be invested in fostering individuals’ critical judgment of health information on the Internet in general, and on PRWs in particular.

In addition to education level and gender, age plays a significant role in individuals’ judgment of what is important to identify a good physician and can be judged after a doctoral visit. Overall, with an increase in age, individuals perceive aspects of decision making with the physician and convenience (reachability, scheduling, etc) more important. Furthermore, older individuals attribute themselves a higher ability to assess physicians’ technical skills. Most likely, older individuals have throughout their lifetime collected hands-on health care experiences that make them more comfortable about their skill to assess physicians. The interpretation of these results nevertheless calls for caution, as potential confounding factors have not been included in the analysis.

### Limitations

The study has a number of limitations. First, there are limitations caused by the recruitment and composition of the two samples. The Delphi study faced a participant dropout rate from n=29 to n=19 from the first to the final round of the study. An additional limitation is posed by the health care consumer sample recruited in this study. The data were collected via snowball sampling on the Internet. Hence, the results cannot claim representativeness. Because the sample had a large share of younger, highly educated females who filled in the questionnaire, it would be recommendable to replicate this study in a different context or country with a more balanced sample.

Also, the results of the comparison between physicians and health care consumers are limited because the Delphi study sample was substantially smaller than the health care consumer sample due to the design (Delphi vs cross-sectional survey). Hence, it was not possible to conduct parametric tests to identify whether the assessments by health care consumers and physicians was statistically significant due to the diverse samples. In order to adjust for this limitation, strict grouping criteria and thresholds, as explained in the methods section, were applied to classify the indicators into the above-listed 4 categories. Furthermore, the cross-sectional survey consisted only of items that were retained after the first round of the Delphi study in order to shorten the survey for health care consumers. Hence, only items that physicians had identified as “important” in the first round of the Delphi study were presented to health care consumers, thereby potentially limiting the variance in the findings.

In addition, the way we assessed individuals’ self-perceived capability to assess a physician’s skill does not allow for definite answers about how well individuals would in reality be able to assess the quality of their health care. Also, it is debatable whether individuals would refrain from judging a physician’s technical ability if they had the chance to do so, even if they indicated previously that they did not think they could assess this aspect of health care. Rather than asking individuals about their perceived ability to assess certain aspects of health care, showing them cases or video samples of treatment encounters and asking them to evaluate and review them could provide additional answers about individuals’ perceived ability to assess their physicians’ performance.

Four specializations with varying skill requirements, client groups, surgical involvement, and payment schemes (pediatricians, orthopedic surgeons, general practitioners, dentists) were invited to participate. In future research, other medical specialists should be invited to develop indicators to identify a good physician separately and only for that particular medical expertise. Indicators of how a good physician can be identified may vary depending on the specialization of physicians studied.

### Conclusions

Physicians’ and health care consumers’ moderate agreement on important and assessable aspects of health care quality suggests that PRWs may profit from presenting information and word of mouth about the quality of a doctor in selected ways. Patients and physicians agreed that health care consumers’ assessment of their provider should be constrained to matters of infrastructure, organization, staff, and his or her interpersonal skills. Technical expertise and outcomes of care were also identified to be important but both physicians and health care providers did not attribute to patients the capability to accurately assess them. Furthermore, our results show that sociodemographic characteristics (age, gender, educational level) play a role in health care consumers’ assessment of what is important to identify a good doctor and what can be evaluated after the first visit.

Our findings suggest that health care consumers may consent to a mixed model in which search and experience aspects of care could be assessed by health care consumers, whereas technical care information could be provided by a source or committee that is competent to asses a physicians’ medical skill (eg, an external expert committee). This could yield a hybrid model in which both health care consumers and experts may contribute information that is adjusted to their level of expertise. How a mixed format of health care consumers’ evaluation of physicians and expert information could best be implemented, and to what extent PRW users would support and use such mixed PRW formats should be the subject of future research.

## Appendix

Multimedia Appendix 1 Indicator comparison in terms of importance of the indicators as assessed by health care consumers and physicians.

Multimedia Appendix 2 Indicator comparison between health care consumers and physicians in terms of health consumers’ evaluation capability.

## Abbreviations

PRW: physician rating website
eWOM: electronic word-of-mouth
QBM: Qualitäts-Basis Modul (Verdag)
