# Supplementary material for: Physician Rating Websites: What Aspects Are Important to Identify a Good Doctor, and Are Patients Capable of Assessing Them? A Mixed-Methods Approach Including Physicians’ and Health Care Consumers’ Perspectives
Source: J Med Internet Res. 2017 May 1;19(5):e127. doi: 10.2196/jmir.6875 (PMC5432667; doi:10.2196/jmir.6875)
Supplement: Multimedia Appendix 1 [file jmir_v19i5e127_app1.pdf]

Appendix 1: Indicator comparison in terms of importance of the indicators as assessed by health care consumers and physicians.

		Health care consumers					Physicians				
Dimension	Indicator	Not important n (%)	Neutral or undecided n (%)	Important n (%)	Mean (SD)	Cate-gory[a]	Not important n (%)	Neutral or undecided n (%)	Important n (%)	Mode	Cate-gory[a]
1 Infrastructure	1.1 The physician's practice is easily accessible and reachable by public transport and car	47 (22.3)	32 (15.2)	132 (62.5)	3.53 (1.24)	2	3 (15.8)	1 (5.3)	15 (78.9)	5	1
	1.2 The physician has necessary instruments to make the diagnosis in the practice	9 (4.3)	43 (20.4)	159 (75.4)	4.00 (.83)	1	3 (15.8)	3 (15.8)	13 (68.4)	4	1
	1.3 The patient's privacy is guaranteed	5 (2.4)	26 (12.3)	180 (85.5)	4.5 (.80)	1	0	0	19 (100)	5	1
	1.4 The practice is clean and hygienic	0	8 (3.8)	203 (96.2)	4.76 (.51)	1	0	0	19 (100)	5	1
2 Staff	2.1 An appropriate number of expert staff is present in the practice (to welcome patients, assist with treatments, etc.)	25 (11.8)	57 (27)	129 (61.1)	3.6 (.95)	1	0	2 (10.5)	17 (89.5)	4	1
	2.2 The medical practice assistants are helpful and friendly	7 (3.3)	17 (8.1)	187 (88.6)	4.36 (.80)	1	0	2 (10.5)	17 (89.5)	5	1

	2.3 The medical practice assistants are experienced in their work	41 (19.4)	63 (29.9)	107 (50.7)	3.39 (1.02)	2	5 (26.3)	4 (21.1)	10 (52.6)	4	1
	2.4 The physician has a lot of work experience and practices already for a longer time	18 (8.5)	58 (27.5)	135 (64)	3.73 (.88)	1	0	1 (5.3)	18 (94.7)	4	1
	2.5 The physician collaborates well with his team	3 (1.4)	19 (9)	189 (89.6)	4.33 (.72)	1	0	1 (5.3)	18 (94.7)	5	1
	2.6 The physician has completed a good education	11 (5.2)	56 (26.5)	144 (68.2)	3.91 (.89)	1	0	1 (5.3)	18 (94.7)	5	1
3 Organization	3.1 The waiting time until the next available appointment is short	11 (5.2)	41 (19.4)	159 (75.4)	3.85 (1.10)	1	4 (21.05)	4 (21.05)	11 (57.9)	5	1
	3.2 The patients are notified in case of appointment delays or cancellations	27 (12.8)	44 (20.9)	140 (66.4)	4.12 (.87)	1	5 (26.3)	1 (5.3)	13 (68.4)	5	1
	3.3 It is easy to schedule an appointment with the physician	1 (0.5)	13 (6.2)	197 (93.4)	4.00 (.97)	1	0	1 (5.3)	18 (94.7)	5	1
	3.4 The physician is available for phone consultations (before or after the appointment)	15 (7.1)	39 (18.5)	157 (74.4)	4.03 (.92)	1	3 (15.8)	1 (5.3)	15 (78.9)	5	1

4 Technical skills	4.1 The patient is diagnosed correctly	0	4<br>(1.9)	207<br>(98.1)	4.82<br>(.45)	1	0	1<br>(5.3)	18<br>(94.7)	5	1
	4.2 The physician asks the relevant questions and orders the correct tests to reach the correct diagnosis	1<br>(0.5)	6<br>(2.8)	204<br>(96.5)	4.74<br>(.52)	1	0	0	19<br>(100)	5	1
	4.3 The physician proceeds systematically and competently to reach the correct diagnosis	0	8<br>(3.8)	203<br>(96.2)	4.69<br>(.53)	1	0	0	19<br>(100)	5	1
	4.4 The physician presents an appropriate and complete number of treatment options to the patient	2<br>(0.9)	15<br>(7.1)	194<br>(91.9)	4.55<br>(.69)	1	0	3<br>(15.8)	16<br>(84.2)	5	1
	4.5 The physician assesses the patient's handicaps correctly and presents him or her with appropriate information and treatment options	2<br>(0.9)	16<br>(7.6)	193<br>(91.5)	4.52<br>(.67)	1	0	0	19<br>(100)	5	1
	4.6 The physician and his team execute the treatment steps correctly	0	9<br>(4.3)	202<br>(95.7)	4.73<br>(.53)	1	0	0	19<br>(100)	5	1
	4.7 The physician and his team adhere to hygiene guidelines	1<br>(0.5)	5<br>(2.4)	205<br>(97.2)	4.79<br>(.49)	1	0	2<br>(10.5)	17<br>(89.5)	5	1
	4.8 The physician executes the treatment competently	1<br>(0.5)	3<br>(1.4)	207<br>(98.1)	4.75<br>(.49)	1	0	0	19<br>(100)	5	1

	4.9 The physician makes the correct diagnosis timely and initiates the treatment swiftly	1 (0.5)	5 (1.9)	202 (95.7)	4.82 (.46)	1	0	0	19 (100)	5	1
5 Interpersonal skills	5.1 The patient can freely express his symptoms (comfortable conversation climate)	3 (1.4)	6 (2.8)	202 (95.7)	4.68 (.62)	1	0	0	19 (100)	4	1
	5.2 The physician involves the patient adequately into the treatment decision	1 (0.5)	8 (3.8)	202 (95.7)	4.62 (.58)	1	0	0	19 (100)	5	1
	5.3 The physician comprehensibly communicates all important information about the diagnosis and treatment	0	2 (0.9)	209 (99.1)	4.81 (.41)	1	0	0	19 (100)	5	1
	5.4 The physician informs the patient about how to handle his or her illness or disease	1 (0.5)	0	210 (99.5)	4.83 (.41)	1	0	0	19 (100)	5	1
	5.5 The physician motivates the patient to actively take part in the treatment process	4 (1.9)	26 (12.3)	181 (85.8)	4.36 (.78)	1	0	0	19 (100)	5	1
	5.6 The patient's concerns are treated confidentially	4 (1.9)	14 (6.6)	194 (91.9)	4.66 (.70)	1	0	0	19 (100)	5	1
	5.7 The physician treats the patient empathically	2 (0.9)	18 (8.5)	191 (90.5)	4.5 (.69)	1	0	0	19 (100)	5	1
	5.8 Decisions about the course of action are made together with the patient	4 (1.9)	11 (5.2)	196 (92.9)	4.63 (.67)	1	0	0	19 (100)	5	1

6 Outcome	6.1 The state of the patient's health improves, in case this was expected	3 (1.4)	14 (6.6)	194 (91.9)	4.55 (.72)	1	0	1 (5.3)	18 (94.7)	5	1
	6.2 The patient is satisfied with the treatment	3 (1.4)	11 (5.2)	197 (93.4)	4.60 (.65)	1	0	2 (10.5)	17 (89.5)	4	1
	6.3 The patient returns to the same physician for check-ups, etc. (patient loyalty)	3 (1.4)	21 (10)	187 (88.6)	4.44 (.73)	1	0	0	19 (100)	4	1
	6.4 The treatment was efficient (i.e. cost-benefit ratio was accurate)	9 (4.3)	21 (10)	187 (88.6)	4.26 (.85)	1	0	1 (5.3)	18 (94.7)	4	1

[a]Categorization: 1= important; 2= not important
