# Supplementary material for: Physician Rating Websites: What Aspects Are Important to Identify a Good Doctor, and Are Patients Capable of Assessing Them? A Mixed-Methods Approach Including Physicians’ and Health Care Consumers’ Perspectives
Source: J Med Internet Res. 2017 May 1;19(5):e127. doi: 10.2196/jmir.6875 (PMC5432667; doi:10.2196/jmir.6875)
Supplement: Multimedia Appendix 2 [file jmir_v19i5e127_app2.pdf]

Appendix 2: Indicator comparison between health care consumers and physicians in terms of health consumers' evaluation capability.

		Health care consumers					Physicians				
Dimension	Indicator	Not evaluable n (%)	Neutral or undecided n (%)	Evaluable n (%)	Mean (SD)	Cate-gory[a]	Not evaluable n (%)	Neutral or undecided n (%)	Evaluable n (%)	Mode	Cate-gory[a]
1 Infrastructure	1.1 The physician's practice is easily accessible and reachable by public transport and car	8 (3.8)	13 (6.2)	190 (90.0)	4.60 (0.84)	1	0	0	19 (100)	5	1
	1.2 The physician has necessary instruments to make the diagnosis in the practice	20 (9.5)	62 (29.4)	129 (61.1)	3.75 (.93)	1	6 (31.6)	13 (68.4)	0	3	2
	1.3 The patient's privacy is guaranteed	43 (20.4)	50 (23.7)	118 (55.9)	3.62 (1.24)	2	0	0	19 (100)	5	1
	1.4 The practice is clean and hygienic	14 (6.6)	21 (10.0)	176 (83.4)	4.29 (.91)	1	0	0	19 (100)	5	1
2 Staff	2.1 An appropriate number of expert staff is present in the practice (to welcome patients, assist with treatments, etc.)	13 (6.2)	46 (21.8)	152 (72.0)	4.00 (.91)	1	2 (10.5)	0	19 (89.5)	4	1
	2.2 The medical practice assistants are helpful and friendly	2 (0.9)	16 (7.6)	193 (91.5)	4.55 (.68)	1	0	1 (5.3)	18 (94.7)	5	1
	2.3 The medical practice assistants are experienced in their work	53 (25.1)	71 (33.6)	87 (41.2)	3.23 (1.04)	2	15 (78.9)	4 (21.1)	0	1	2

	2.4 The physician has a lot of work experience and practices already for a longer time	21 (10)	77 (36.5)	113 (53.6)	3.57 (.93)	1	3 (15.3)	15 (78.9)	1 (5.3)	3	2
	2.5 The physician collaborates well with his team	23 (10.9)	60 (28.4)	128 (60.7)	3.73 (.99)	1	0	0	19 (100)	4	1
	2.6 The physician has completed a good education	64 (30.3)	69 (32.7)	78 (37.0)	3.15 (1.16)	2	15 (78.9)	4 (21.1)	0	1	2
3 Organization	3.1 The waiting time until the next available appointment is short	6 (2.8)	41 (19.4)	164 (77.7)	4.20 (.96)	1	0	0	19 (100)	5	1
	3.2 The patients are notified in case of appointment delays or cancellations	24 (11.4)	36 (17.1)	151 (71.6)	3.99 (1.10)	1	0	0	19 (100)	5	1
	3.3 It is easy to schedule an appointment with the physician	5 (2.4)	19 (9.0)	187 (88.6)	4.40 (.79)	1	0	0	19 (100)	5	1
	3.4 The physician is available for phone consultations (before or after the appointment)	20 (9.5)	44 (20.9)	147 (69.7)	3.99 (1.10)	1	0	0	19 (100)	5	1
4 Technical skills	4.1 The patient is diagnosed correctly	49 (23.2)	81 (38.4)	81 (38.4)	3.22 (1.06)	2	4 (21.1)	2 (10.6)	13 (68.4)	4	1
	4.2 The physician asks the relevant questions and orders the correct tests to reach the correct diagnosis	37 (17.55	89 (42.2)	85 (40.3)	3.31 (1.01)	2	5 (26.6)	14 (73.7)	0	0	2
	4.3 The physician proceeds systematically and competently to reach the correct diagnosis	30 (14.2)	75 (35.5)	106 (50.2)	3.47 (1.00)	2	4 (21.1)	13 (68.4)	2 (10.5)	0	2

	4.4 The physician presents an appropriate and complete number of treatment options to the patient	37 (17.5)	57 (27.0)	117 (55.5)	3.52 (1.07)	2	3 (15.8)	15 (78.9)	1 (5.3)	0	2
	4.5 The physician assesses the patient's handicaps correctly and presents him or her with appropriate information and treatment options	30 (14.2)	63 (29.9)	118 (55.9)	3.55 (.98)	1	0	1 (5.3)	18 (94.7)	4	1
	4.6 The physician and his team execute the treatment steps correctly	41 (19.4)	81 (38.4)	89 (42.2)	3.34 (1.01)	2	3 (15.8)	15 (78.9)	1 (5.3)	0	2
	4.7 The physician and his team adhere to hygiene guidelines	34 (16.1)	51 (24.2)	126 (59.7)	3.71 (1.13)	1	14 (73.9)	5 (26.6)	0	2	2
	4.8 The physician executes the treatment competently	27 (12.8)	73 (34.6)	111 (52.6)	3.59 (1.00)	2	4 (21.1)	13 (68.4)	2 (10.5)	0	2
	4.9 The physician makes the correct diagnosis timely and initiates the treatment swiftly	42 (19.9)	67 (31.8)	102 (48.3)	3.40 (1.06)	2	0	2 (10.5)	17 (89.5)	4	1
5 Interpersonal skills	5.1 The patient can freely express his symptoms (comfortable conversation climate)	4 (1.9)	21 (10)	186 (88.2)	4.51 (.77)	1	0	0	19 (100)	5	1
	5.2 The physician involves the patient adequately into the treatment decision	6 (2.8)	39 (18.5)	166 (78.7)	4.18 (.85)	1	0	0	19 (100)	5	1
	5.3 The physician comprehensibly communicates all important information about the diagnosis and treatment	8 (3.8)	35 (16.6)	168 (79.6)	4.21 (.87)	1	0	0	19 (100)	5	1

	5.4 The physician informs the patient about how to handle his or her illness or disease	18 (8.5)	46 (21.8)	147 (69.7)	3.92 (.98)	1	2 (10.5)	1 (5.3)	16 (84.2)	1	1
	5.5 The physician motivates the patient to actively take part in the treatment process	9 (4.3)	36 (17.1)	166 (78.7)	4.19 (.87)	1	1 (5.3)	1 (5.3)	17 (89.5)	4	1
	5.6 The patient's concerns are treated confidentially	78 (37)	62 (29.4)	71 (33.6)	3.00 (1.35)	1	1 (5.3)	1 (5.3)	17 (89.5)	5	1
	5.7 The physician treats the patient empathically	6 (2.8)	28 (13.3)	177 (83.9)	4.38 (.82)	2	0	0	19 (100)	5	1
	5.8 Decisions about the course of action are made together with the patient	11 (5.2)	23 (10.9)	177 (83.9)	4.30 (.90)	1	0	3 (15.8)	16 (84.2)	4	1
6 Outcome	6.1 The state of the patient's health improves, in case this was expected	26 (12.3)	44 (20.9)	141 (66.8)	3.80 (1.06)	1	0	0	19 (100)	1	1
	6.2 The patient is satisfied with the treatment	9 (4.3)	28 (13.3)	174 (82.5)	4.32 (.88)	1	0	1 (5.3)	18 (94.7)	5	1
	6.3 The patient returns to the same physician for check-ups, etc. (patient loyalty)	22 (10.4)	27 (12.8)	162 (76.8)	4.10 (1.10)	2	0	0	19 (100)	5	1
	6.4 The treatment was efficient (i.e. cost-benefit ratio was accurate)	60 (28.4)	67 (31.8)	84 (39.8)	3.18 (1.06)	1	0	16 (84.2)	3 (15.8)	0	2

[a]Categorization: 1= evaluable; 2= not evaluable
